# Criminal Abortion in Japan

**Published:** 1876-08

**Authors:** 


CRIMINAL ABORTION IN JAPAN.—The higher classes consider it a disgrace, besides it is forbidden by their laws. Nevertheless it is openly practiced on women of the lower class. The method, usually employed since olden times, has only been adopted by European obsetricians during the present century. A piece of the root of achyranthes aspera, more than one foot long, about as thick as a quill, and very flexible, is introduced between the uterus and the fœtal membranes, where it is retained for a day or two. The root is smeared with musk and then introduced by the aid of two fingers into the vagina. Besides, musk is given internally. The result is always certain.

A modification of this method consists in the introduction of silk thread impregnated with musk. Aside from this pieces of pointed wood are introduced into the os, which method is not seldom followed by death. The Japanese regard the fourth and fifth month of pregnancy as the best time for the operation.—*Detroit Review of Med. and Pharm.* Feb., 1875; also *Virchow's Archives*, Dec., 1874.

